# Extraction of gray-scale intensity distributions from micro computed tomography imaging for femoral cortical bone differentiation between low-magnesium and normal diets in a laboratory mouse model

**DOI:** 10.1038/s41598-019-44610-8

**Published:** 2019-05-31

**Authors:** Shu-Ju Tu, Shun-Ping Wang, Fu-Chou Cheng, Ying-Ju Chen

**Affiliations:** 1 Department of Medical Imaging and Radiological Sciences, College of Medicine, Chang Gung University, Tao-Yuan, Taiwan; 2 Department of Medical Imaging and Intervention, Linkou Chang Gung Memorial Hospital, Tao-Yuan, Taiwan; 3 Department of Orthopedics, Taichung Veterans General Hospital, Taichung, Taiwan; 4 College of Science, Tunghai University, Taichung, Taiwan; 5 Stem Cell Center, Department of Medical Research, Taichung Veterans General Hospital, Taichung, Taiwan; 6 Department of Food and Nutrition, Providence University, Taichung, Taiwan

**Keywords:** Preclinical research, Biomedical engineering

## Abstract

Previous studies have shown that the geometric development of femoral trabecular bone is affected by insufficient dietary intake of magnesium. However, it is not clear whether the development of femoral cortical bone can be quantitatively evaluated according to a diet with inadequate magnesium supplementation. Therefore, we used a micro computed tomography (CT) imaging approach with a laboratory mouse model to explore the potential application of texture analysis for the quantitative assessment of femoral cortical bones. C57BL/6J male mice were divided into two groups, where one group was fed a normal diet and the other group was fed a low-magnesium diet. We used a micro CT scanner for image acquisition, and the subsequent development of cortical bone was examined by texture analysis based on the statistical distribution of gray-scale intensities in which seven essential parameters were extracted from the micro CT images. Our calculations showed that the mean intensity increased by 7.20% (*p* = 0.000134), sigma decreased by 29.18% (*p* = 1.98E-12), skewness decreased by 19.52% (*p* = 0.0000205), kurtosis increased by 9.62% (*p* = 0.0877), energy increased by 24.19% (*p* = 3.32E-09), entropy decreased by 6.14% (*p* = 3.00E-10), and the Nakagami parameter increased by 104.32% (*p* = 4.13E-12) in the low-magnesium group when compared to the normal group. We found that the statistical parameters extracted from the gray-scale intensity distribution were able to differentiate between femoral cortical bone developments in the two different diet groups.

## Introduction

Bones are critical organs that protect internal organs and are the major mineral reservoir of the body. The musculoskeletal system includes bones, muscles, and joints. The functions of these different tissues are movement and biomechanical performance. Fracture risk is highly correlated with the bone strength of femoral cortical bones^1–4^. In recent years, micro computed tomography (CT), which is an x-ray based imaging technology with high spatial-resolution, has been successfully used to quantify bone quality in different animal experiments^1–4^.

The current approach for femoral cortical bone assessment includes quantitative assessment of bone mineral density (BMD) and bone morphology^1–4^. These parameters are calculated based on a user-defined region of interest (ROI) in bone images. They are considered a single quantity to represent a global average with a specific physical attribute for the entire ROI. Unfortunately, the histogram distribution of gray-scale intensities obtained from images has not yet been investigated. In particular, the statistical distribution of gray-scale intensities may be critical for the evaluation of local variations in the ROI^5,6^.

In cancer imaging research, a similar texture approach of gray-scale intensity distribution in CT images has been used to quantify tumour heterogeneity^7^. Previous studies have shown that some texture parameters derived from the gray-scale intensity distribution such as entropy and energy are highly correlated with tumour angiogenesis and metabolism^8^. In previous studies, bone heterogeneity has been shown to be associated with fracture resistance^9–11^. In particular, bone heterogeneity may be the result of the composition of different bone tissues, bone turnover dynamics between osteoblasts and osteoclasts, or bone mineralization kinetics^12–15^. In CT imaging physics, attenuation of x-ray transmission to matter corresponds to the material composition^16^. Consequently, it is highly conceivable to anticipate a close relationship between bone heterogeneity and gray-scale intensity variations between adjacent pixels in CT images.

**Figure 1:**
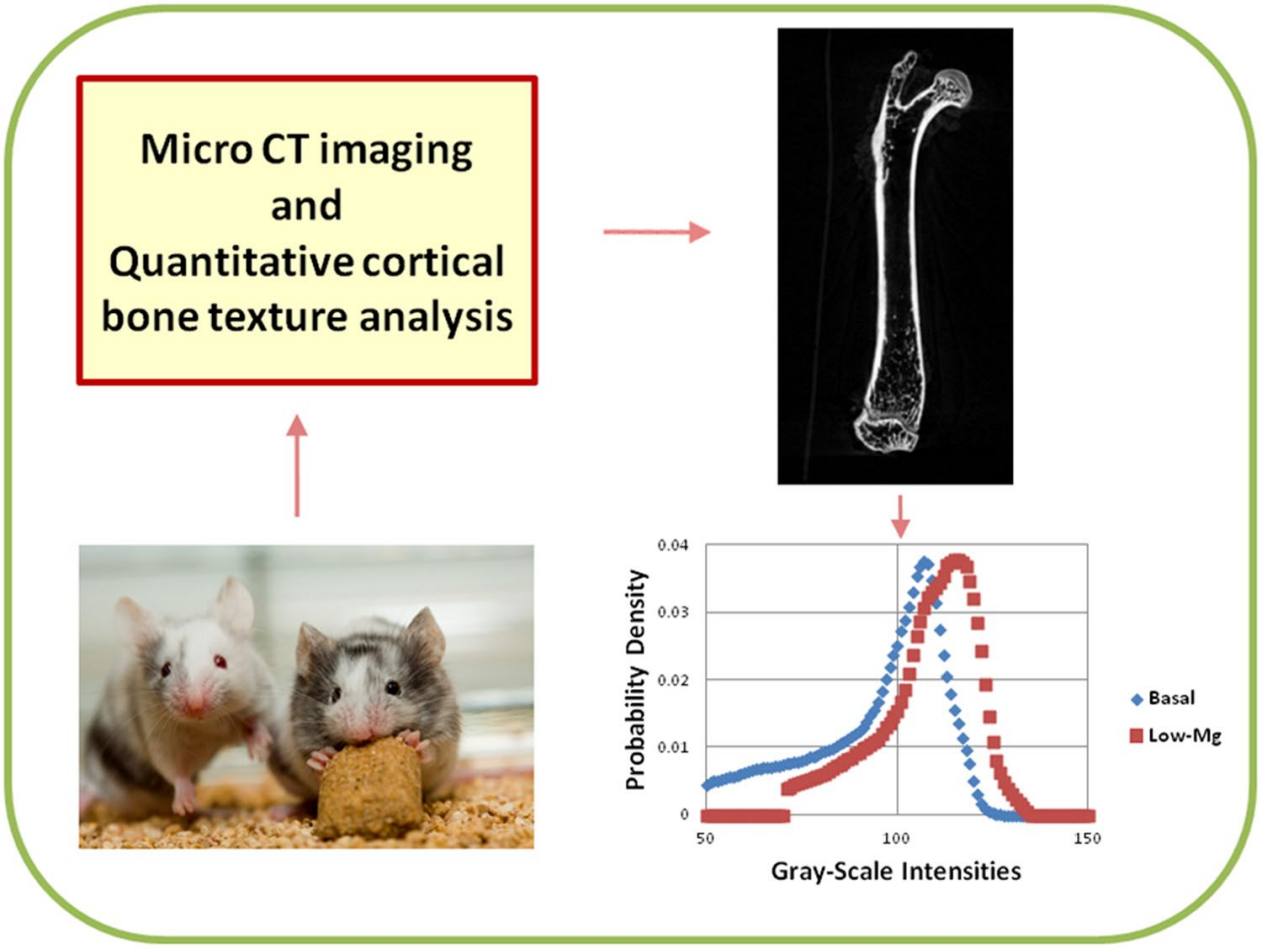
A flowchart of our technical approach to bone assessment in laboratory mice with micro CT imaging and quantitative analysis of gray-scale intensity distribution.

Osteoporosis is an important health problem for the elderly. There is a growing consensus regarding the role of microarchitecture in osteoporotic bone loss and fragility. This trend has been promoted by advances in imaging technology, which have enabled a transition from assessments of mass to microarchitecture. In previous studies, imaging of trabecular bone has been a research focus, but advances in resolution have also enabled the detection of cortical bone micro-architecture. However, some reports have shown that cortical bone is intimately linked to the remodelling process, which underpins bone loss; thus, our fundamental understanding of bone health through imaging animal models could be potentially improved. Therefore, one main goal of our study was to use magnesium-deficient animals and micro CT imaging to develop a new method for cortical bone analysis. It has been shown that the quality of trabecular bone development is inferior in mice fed a diet with an insufficient amount of magnesium, compared with mice fed a normal diet^17–19^. In this study, we examined whether the quantitative texture analysis of gray-scale intensity distribution obtained from micro CT images is effective for cortical bone assessment in laboratory mice. A summary of this study is shown in Fig. 1.

## Materials and Methods

### Animal model

C57BL/6J male mice (4 weeks of age) were purchased from BioLASCO Taiwan Co., Ltd. (Taipei, Taiwan) and housed in a temperature-controlled (22 °C) and light-controlled room (12:12 h light-dark cycle). Animal care and experimental procedures were approved by the Institutional Animal Care and Use Committee of Taichung Veterans General Hospital (ID: La-1021102). Three R (3Rs) were included in the affidavit of the approved animal use protocol. Animal experiments were performed in accordance with relevant guidelines and regulations. All animals were supplied with a basal diet over a week long acclimatization period. Subsequently, mice were randomly assigned to two groups, a control group (n = 7) and a low magnesium group (n = 7), and were fed either the basal diet (5755 TestDiet containing 0.7 mg/g Mg, Richmond, IN, USA) or a low magnesium diet (5865 TestDiet containing <0.08 mg/g Mg), respectively, for 8 weeks. The mice were fed their respective diets and distilled water ad libitum. After 8 weeks on the diets, the experimental animals were sacrificed by carbon dioxide asphyxiation, and the femoral bones were collected from each mouse within 20 minutes. All removed samples were wrapped with gauze moistened with 0.9% saline solution and then preserved at −20 °C. Then, each femoral bone was scanned by micro CT for quantitative assessment.

### Micro CT imaging system

The imaging system was a desk-top micro CT scanner (SkyScan 1076, Bruker Micro CT, Belgium) in which the x-ray tube and detector were housed and integrated in a radiation-shield instrument. The x-ray beam was collimated as a cone beam irradiation system. The detector of x-ray was a charge-coupled device camera with 11-million pixels. The anode voltage range was between 20 and 100 kV. A modified and high-speed Feldkamp algorithm was used in for image reconstruction. The scanning bed for the animal is made of carbon fibre material. In our work, micro CT scanner was operated at 50 kV, and a 0.5 mm thickness aluminum filter was used for optimal image contrast. The current setting was 0.2 mA and the total detector exposure time was 1440 seconds. A total of 720 project images were acquired through circular scanning with a total of 720 rotational steps. The distance from the source to the object was 121 mm, and the distance from the source to the camera detector was 165 mm. Images were reconstructed and processed with a spatial resolution of 9.0 µm. The range of attenuation coefficients used in the reconstruction software was between 0 and 0.15. The options of beam hardening and ring artefact correction were selected as 40% and 10.

Several clinical bone imaging systems are available from different vendors among different healthcare institutions. Additionally, a single machine may have multiple settings for different applications of bone imaging. It is thus difficult to make a correct bone disorder diagnosis, if the images are acquired using different systems and settings. For example, bone mineral density from dual-energy x-ray absorptiometry imaging and parameters from quantitative CT cannot be used to compare clinical changes in bone disease in the same patient if those instruments are from different vendors or use different setting. To test our methodology of texture analysis, we therefore used identical settings in a single micro CT scanner for the two animal groups. These settings included all scanning and image reconstruction parameters.

### Region of interest

The mid-plane at the mid-diaphysis of the femoral cortical bone was located and a length of 0.5 mm along the direction of the long-axis was then delineated as the ROI^1^. CTAn (CT-Analyser, Bruker micro CT, Belgium) and Avizo (Visualization Sciences Group, Massachusetts, USA) were used to perform image analysis. The final sets of ROIs were approved by an experienced radiologist specialized in musculoskeletal imaging. The ROI, based on suggestions from previous literature, provided us with a sufficient pixel number for statistical evaluation^1,20^. We chose this ROI because it was an area where the theoretical biomechanical bone strength could be evaluated and where we can use a material testing system to measure the experimental bone strength. In particular, our analysis of the gray-scale intensity distribution for this ROI could be subsequently compared and correlated with theoretical biomechanical strength calculations and experimental bone strength measurements by a material testing system.

### Quantitative parameters

In this study, we used a methodology based on the statistical distribution of the gray-scale intensities to extract image features from micro CT images for the assessment of bone development in our animal experiments. The gray-scale intensities were directly obtained from the pixels of micro CT images. The intensities are positive integers with values between 0 and 255. Quantitative parameters were computed from the distribution of the gray-scale intensities in the pixels of ROI. In our work, the computed quantities were mean, sigma, skewness, kurtosis, energy, entropy, and the Nakagami parameter^16,21^. The following equations were used in our calculations:

1 $${\rm{Mean}}=\bar{m}=\sum _{k=1}^{n}\,k\times {P}_{k}$$

2 $${\rm{Sigma}}={\rm{\sigma }}={[\sum _{k=1}^{n}{(k-\bar{m})}^{2}\times {P}_{k}]}^{\frac{1}{2}}$$

3 $${\rm{Skewness}}=\frac{1}{{\sigma }^{3}}\sum _{k=1}^{n}\,{(k-\bar{m})}^{3}\times {P}_{k}$$

4 $${\rm{Kurtosis}}=\frac{1}{{\sigma }^{4}}\sum _{k=1}^{n}\,{(k-\bar{m})}^{4}\times {P}_{k}-3$$

5 $${\rm{Energy}}=\sum _{k=1}^{n}\,{\lceil {P}_{k}\rceil }^{2}$$

6 $${\rm{Entropy}}=\sum _{k=1}^{n}\,{P}_{k}\times {\mathrm{log}}_{2}{P}_{k}$$

7 $${\rm{Nakagami}}\,{\rm{parameter}}=\frac{{{\rm{E}}}^{2}({X}^{2})}{{\rm{Var}}({X}^{2})}$$

where *k* is an integer value of the gray-scale intensity between 0 and 255, *P*_*k*_ is the height of normalized distribution of the gray-scale intensity *k*, E(*X*) is the expectation value of *X*, Var(*X*) is the variance of *X*, and *X* is the variable of the gray-scale intensity^21,22^. A short summary of these parameters is listed in Table 1.

**Table 1:** A summary of parameters used in our quantitative analysis of gray-scale intensity distributions for femoral cortical bones with micro CT imaging.

Parameters	Definition and description
Mean	A first-order statistical quantity and the average gray-scale intensity of the image pixels in the region of interest.
Sigma	The standard deviation and a second-order statistical quantity for variation quantification.
Skewness	A third-order statistical quantity and measure of asymmetry in the distribution. A Gaussian distribution is symmetric at the peak and has a skewness value of zero. A positive skewness represents the central population tendency of the distribution shifted to the left. A negative skewness represents the central population tendency of the distribution shifted to the right.
Kurtosis	A fourth-order statistical quantity and measure of the peakedness of the distribution. A positive kurtosis represents a distribution of a relatively peaked shape. A negative kurtosis represents a distribution of a relatively flat shape.
Energy	An image feature that represents variations and uniformity of gray-scale intensities in images.
Entropy	An image feature that represents information of randomness, complexity, or heterogeneity.
Nakagami parameter	An image feature that represents the scatter characteristics and microstructure of the tissue.

### Bone mineral density phantom

In clinical medicine, BMD is an essential reference for the diagnosis of osteoporosis and the potential risk of bone fracture^23,24^. To further investigate whether one of our investigated parameters was correlated with BMD, we used a standard BMD phantom (QRM-microCT-HA, QRM GmbH, Moehrendorf, Germany) to acquire micro CT images and calculated the corresponding parameters^25^. The phantom consists of five cylindrical inserts of known densities of calcium hydroxyapatite (Ca-HA), Ca_10_(PO_4_)_6_(OH)_2_. Proprietary epoxy resin is uniformly filled as the base material. The BMD values of the cylindrical inserts are 200, 400, 600, 800 and 1000 mg-HA/cm^3^. The physical size of the cylindrical insert was 5 mm in diameter and 38 mm in length.

### Statistical analysis

Microsoft Excel and Medcalc software (version 11.4, Mariakerke, Belgium) were used for statistical analysis. Data are presented as the mean ± standard deviation. A t-test was performed for statistical analysis. An F-test was used to determine whether an equal-variance assumption should be applied. A *p*-value was used to determine whether the difference between two groups was statistically significant. We considered a result to be significantly different when the *p*-value was less than 0.05.

## Results and Discussion

**Figure 2:**
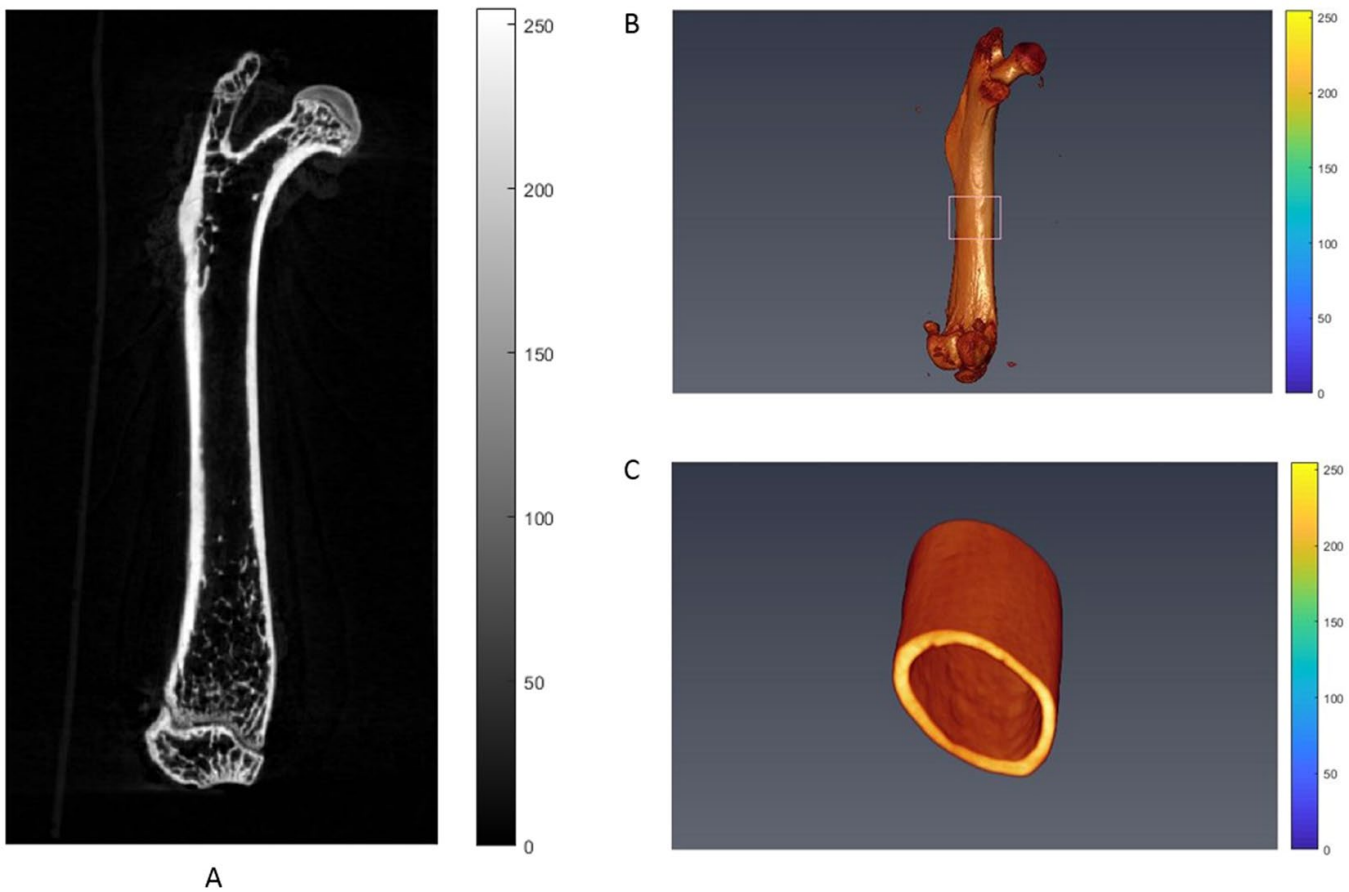
(**A**) An image of a coronal view of a femur bone acquired from the micro CT scanning. The region of interest is shown in the added box. (**B**) A 3D volume-rendered image of a femur bone acquired from the micro CT scanning. The region of interest is shown in the added box. (**C**) A 3D volume-rendered image of the region of interest.

**Figure 3:**
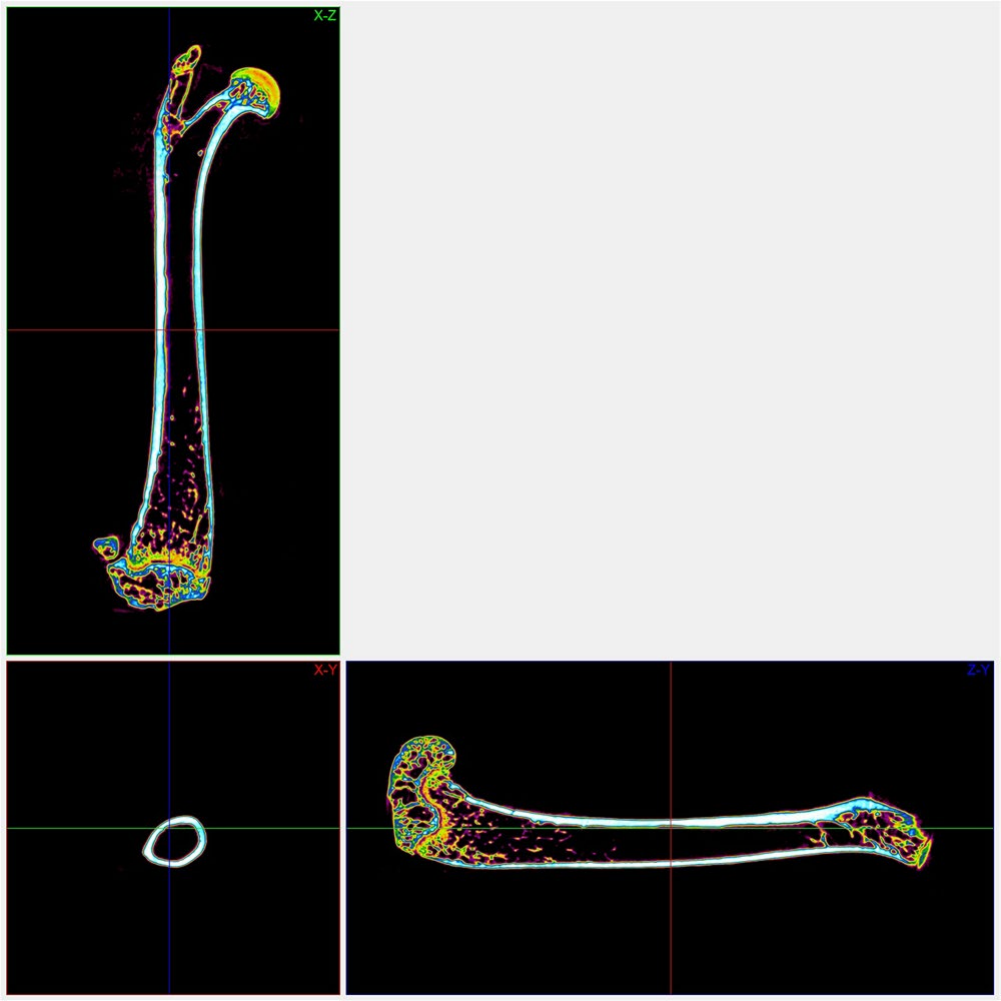
The corresponding images of pseudo-colour presentation with three views from different planes.

**Figure 4:**
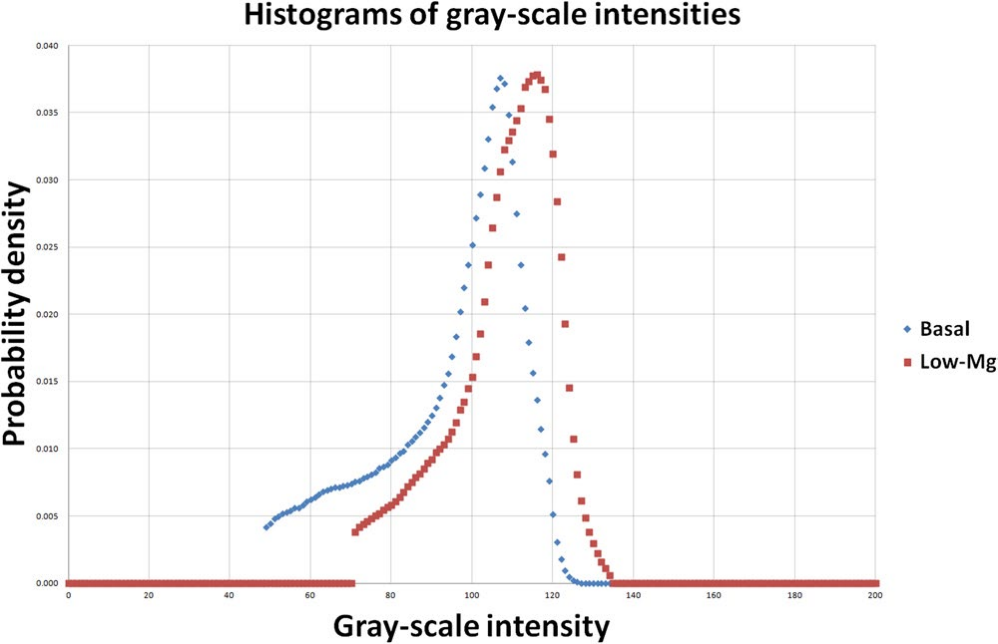
Distributions of gray-scale intensities extracted from micro CT imaging of femoral cortical bones.

A micro CT image of a femoral cortical bone, depicted in gray-sale intensities, and its region of interest are shown in Fig. 2A–C. The corresponding images of pseudo-colour presentation with views from three different planes are included in Fig. 3. The distributions of gray-scale intensities extracted from micro CT images for different groups are shown in Fig. 4. The results of the associated quantities derived from the distribution are listed in Table 2. We observed that the mean pixel intensity increased by 7.20% (*p* < 0.005), sigma decreased by 29.18% (*p* < 0.005), skewness decreased by 19.52% (*p* < 0.005), kurtosis increased by 9.62% (*p* < 0.005), energy increased by 24.19% (*p* < 0.005), entropy decreased by 6.14% (*p* < 0.005), and the Nakagami parameter increased by 104.32% (*p* < 0.005) in the group of mice with the low magnesium diet compared to the respective parameters in the mice with the control diet.

**Table 2:** Quantitative assessment of femoral cortical bones at mid-diaphysis with our parameters.

Parameters	Basal diet	Low-Mg diet	*p*-value
	Average	Average	
Mean	98.1 ± 1.4	105 ± 1	0.000134
Sigma	17.8 ± 0.1	12.6 ± 0.1	1.98E-12
Skewness	−0.906 ± 0.018	−0.729 ± 0.021	0.0000205
Kurtosis	−0.0606 ± 0.0507	−0.0665 ± 0.0431	0.0877
Energy	0.0201 ± 0.0003	0.0250 ± 0.0002	3.32E-09
Entropy	4.10 ± 0.01	3.85 ± 0.01	3.00E-10
Nakagami parameter	9.54 ± 0.33	19.5 ± 0.2	4.13E-12

These parameters are derived from the gray-scale intensity distribution in micro CT images. We consider the parameter useful to differentiate between the two experimental groups when the *p*-value is less than 0.05.

There are limitations of this texture approach that was used to quantify cortical bone developments between different diet groups. Our technical approach extracted quantitative parameters from the statistical distribution which was obtained from gray-scale intensities in the micro CT images. However, there are factors which could potentially affect the results of these quantitative parameters. These factors included imaging settings of micro CT acquisitions, image noise, image voxel number, image reconstruction algorithms, image artifacts, and post image-processing algorithms. Some potential sources responsible for the image noise are the quantum noise from the photon counting statistics, electronics devices, and the size of image voxel. There are three options for the voxel size in the micro CT system. These options were 9, 18, and 35 µm. The size of image voxel would determine the total number of voxels within the volume of femoral cortical bone. The gray-scale intensities in all image voxels of femoral cortical bone were used to build the statistical distribution. Then the quantitative parameters are derived from these statistical distributions. In this study, the voxel size of 9.0 µm was selected for image reconstruction. This selection allowed us to use the largest number of image voxels in cortical bone images to represent the gray-scale intensity distribution. There are reports that discuss the effect of image quality and image settings on texture analysis^26^. Nevertheless, in our study, imaging settings were identical for the two groups of mouse specimens. The results should allow us to differentiate between the two different diet groups of mice.

**Figure 5:**
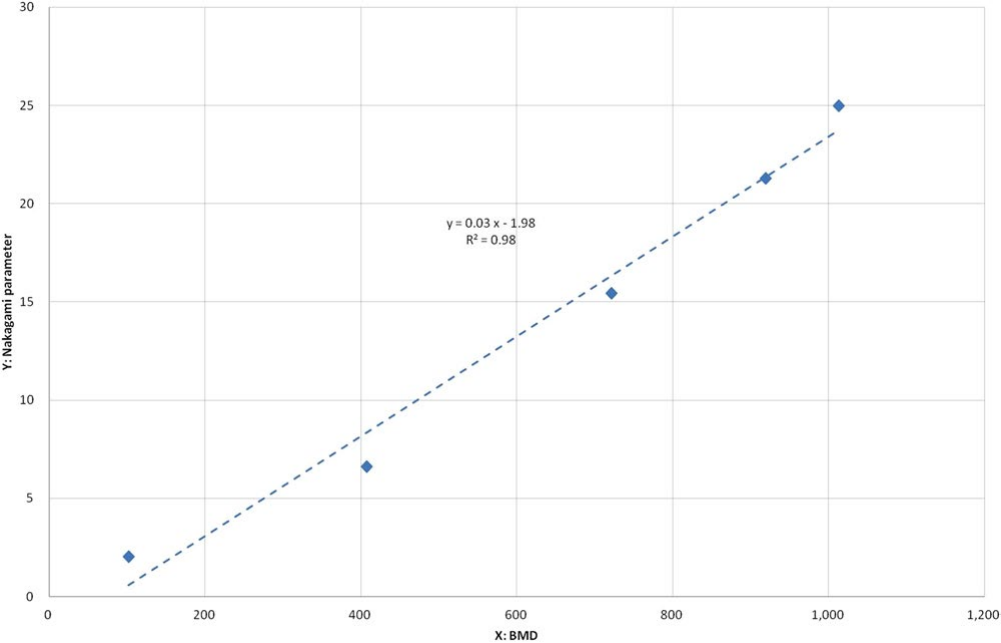
A linear correlation was identified between the Nakagami parameter and bone mineral density with a standard micro CT phantom with five inserts of different calibrated bone mineral densities.

BMD is an essential indicator for osteoporosis and a potential risk assessor of bone fracture^23,24^. A BMD phantom for micro CT imaging was used to study whether one of our investigated parameters was correlated with the quantity of BMD. The Nakagami parameter was highly correlated with BMD value, as shown in Fig. 5 (r^2^ = 0.979, *p* = 0.00121).

In addition to cortical bones, femoral bones are rich in trabecular bones. Trabecular bone consists of tiny bone tissue units that are densely interconnected^23,24,27^. In x-ray imaging, these tiny trabecular bone units induce partial volume effect on micro CT images^16^. Consequently, the gray-scale intensities may substantially decrease in pixel value at locations with high concentrations of trabecular bone. Therefore, our approach of using gray-scale intensity distributions may not be useful for trabecular bone analysis^4,19^. However, femoral cortical bones are dense and compact. The image artifact of partial volume is minimal. The biomechanical strength of femoral cortical bones is directly responsible for the risk assessment of bone fracture. The primary purpose of our manuscript was to report our new approach to bone quality evaluation between groups with two different diets. Therefore, femoral cortical bones were used in our study.

**Figure 6:**
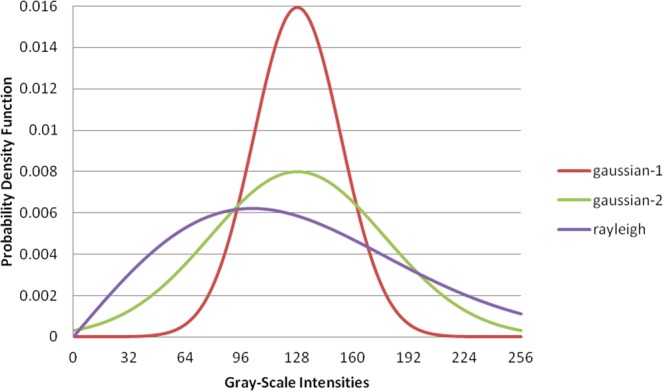
Three different probability distribution functions with the same mean of 128. These probability distribution functions are normalized to 1. Although the means are identical in these functions, the relative percentages of low-density populations in these functions are different.

Orthopaedic surgeons and radiologists use the BMD index for key assessment of bone strength and treatment response. However, the BMD value mainly represents an average quantity for the delineated ROI and it may be critical to further evaluate the statistical histograms of gray-scale intensities in images^12–15^. In particular, the average gray-scale intensity can be obtained from the gray-scale intensity distribution, but it is unlikely that a correct statistical distribution could be formalized based on a single average^28,29^. Additionally, identical averages could be obtained from different distributions, as illustrated in Fig. 6. In particular, images with different distributions of gray-scale intensities could suggest different bone strength profiles.

Dual-energy x-ray absorptiometry and the quantity of bone mineral density have been used for related osteoporosis diagnosis in current clinical bone diagnoses. However, the image of dual-energy x-ray absorptiometry is limited to a two-dimensional radiograph and the BMD quantity, an average of the entire defined area, cannot fully reflect bony health and strength. Compared to BMD, bone quality such as bone microarchitectures, the composition of bone tissue, the degree of mineralization, and bone morphology, is considered more crucial to bone strength. In recent progresses of bone imaging, x-ray based CT systems such as quantitative CT with low radiation exposure have been used for advanced diagnoses of bone disorders^30–32^. The morphology of femoral trabecular bones can be quantified from these CT images. Additionally, three-dimensional images allow us to explore volumetric bone information. In our study which was based on an animal model, we extracted a series of quantitative parameters from gray-scale intensity distributions of micro CT images to differentiate femoral cortical bone strength between two groups of mice with different diets. In addition to the index of bone mineral density from trabecular bones, our texture analysis study using a series of quantitative parameter extractions from femoral cortical bones can potentially be applied to further bone strength assessment with a 3D bone scanning system such as the quantitative CT. Therefore, we propose using a 3D bone imaging system such as a quantitative CT to build a bone informatics system for a general healthy population with our quantitative parameters and other useful quantities. For further clinical applications, more extensive research is needed to study the potential correlations between these texture parameters, mechanical bone strength, bone histology, and chemical composition analysis of cortical bones.

In the NRecon software of our micro CT system (SkyScan 1076, Bruker Micro CT, Belgium), we were able to choose the range of attenuation coefficients for bone image reconstruction. The choice of attenuation coefficient range allows users to visualize bone images with better contrast between hard and soft tissues. However, the choice of attenuation coefficient range affects the gray-scale intensities in the image pixels where the gray-scale intensities are integers distributed between 0 and 255. Therefore the results of texture analysis depended on the choice of the attenuation coefficient range. It is thus essential to keep an identical set of scanning settings and reconstruction parameters for imaging different animal groups. In this study, we used identical reconstruction parameters and imaging settings for comparing the texture analysis results between animals with different diets.

As shown in Fig. 6, an identical mean can be obtained from three different probability distribution functions:

8 $${f}_{1}(x)=\frac{1}{25\times \sqrt{2\pi }}{e}^{-0.5\times \frac{{(x-128)}^{2}}{{25}^{2}}}$$

9 $${f}_{2}(x)=\frac{1}{50\times \sqrt{2\pi }}{e}^{-0.5\times \frac{{(x-128)}^{2}}{{50}^{2}}}$$

10 $${f}_{3}(x)=\frac{x}{{100}^{2}}{e}^{-0.5\times \frac{{x}^{2}}{{100}^{2}}}$$

where *x* ∈ [0, 255], and *f*_1_(*x*), *f*_2_(*x*), and *f*_3_(*x*) are normalized to 1. If the probability distribution functions represent the distribution of gray-scale intensities of micro CT images for bone analysis, then 0.55%, 9.69%, and 18.89% of gray-scale intensities in the functions *f*_1_(*x*), *f*_2_(*x*), and *f*_3_(*x*), respectively, are below the 25th percentile (the first quartile), even though these three distribution functions have identical means of 128. A high percentage of low gray-scale intensity in micro CT images indicates a high percentage of bone volume with low density; therefore, this could suggest an enlargement of areas with low bone density or an increase in the risk of bone fracture.

**Figure 7:**
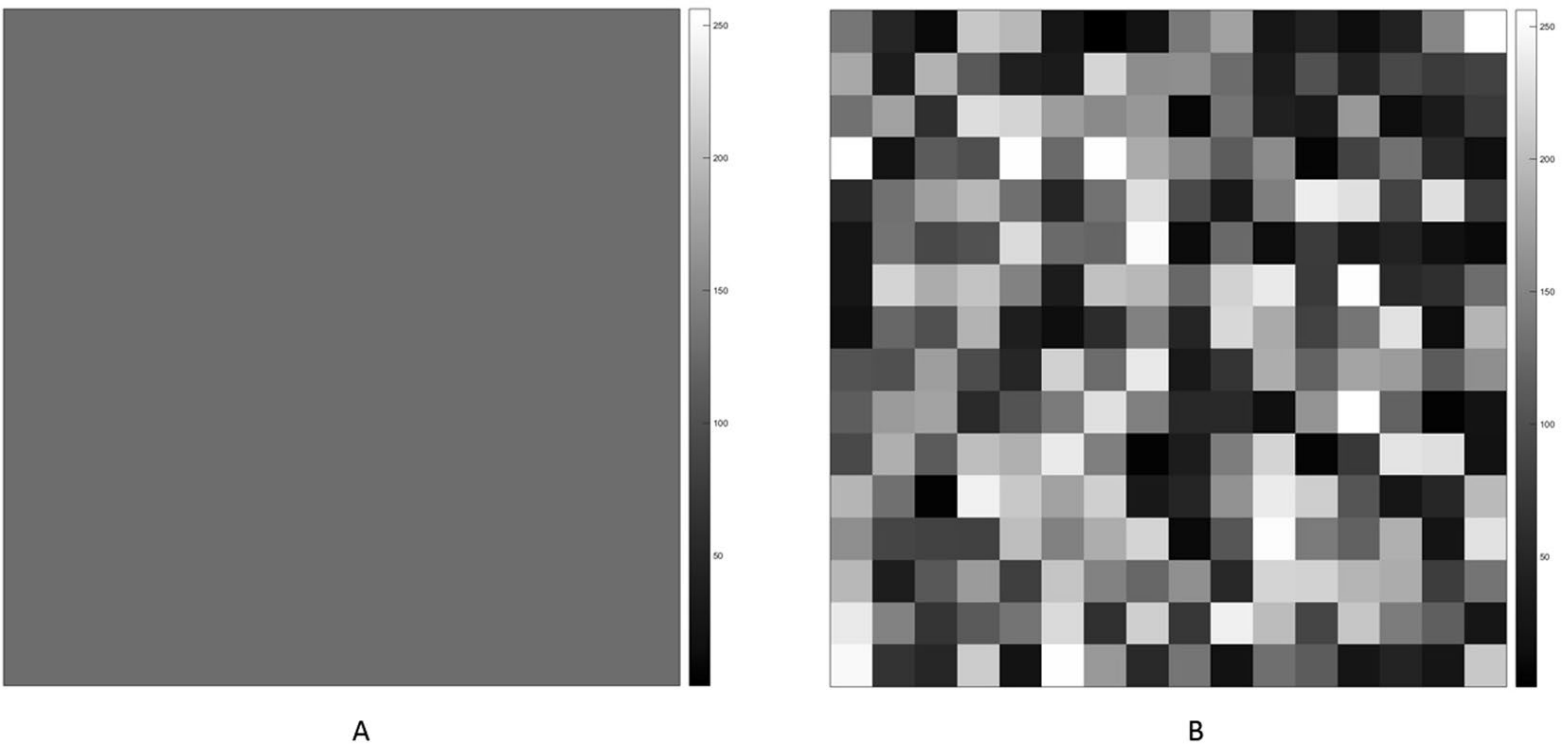
(**A**) An 8-bit image (16 by 16) with a uniform distribution. The mean is 128 and the gray-scale intensities in the pixel are set to 128. The gray-scale intensity is corresponded to the relative bone density. (**B**) An 8-bit image (16 by 16) with a non-uniform distribution. The mean is 128 and the gray-scale intensities in the pixel are generated by a random number generator. The gray-scale intensity corresponds to the relative bone density. The images were produced by MATLAB.

To further demonstrate the potential effect of variations in gray-scale intensities between pixels, we proposed two scenarios as shown in Fig. 7, in which 8-bit images are depicted with gray-scale intensities from 0 to 255. These figures show different distributions of gray-scale intensities in 16 × 16 pixel space, while the means of the two figures are identical. The gray-scale intensity is identical for all pixels in Fig. 7A, while pixels with low and high intensity values are randomly distributed in Fig. 7B. The boundary between pixels with different densities represents a discontinuity interface, a location that is more vulnerable to breakage or fracture^33,34^. Therefore, we may expect the strength of resistance against an external force in the image shown in Fig. 7A to be stronger than that in the image shown in Fig. 7B.

In our analysis of micro CT images, the mean is a first-order statistical parameter and represents the average of gray-scale intensities over the pixels in the delineated ROI^16,21,22^. A large mean in bone images represents high average bone physical density in that area. In this study, the mean value in low-magnesium mice was greater than that in control mice (*p* < 0.005).

In statistics, sigma is a second-order parameter and represents the standard deviation of a sample population^16,21,22^. Sigma is a measure used to quantify the spread over the mean. A distribution with a smaller sigma indicates that the functional shape is sharper around the mean and therefore more data points are centrally populated around the mean value. In our distribution analysis of gray-scale intensities, a small sigma represents high uniformity in the micro CT image, with a large population of pixels with gray-scale intensities similar to the average. A large sigma represents a wide range of gray-scale intensities and hence the bone images look non-uniform. A bone image with a wide gray-scale intensity distribution may suggest that the bone tissue is highly heterogeneous.

In previous studies of bone mineralization density distribution (BMDD), a quantity of peak width was defined to show the correlation of the width of the calcium peak in the BMDD with the outcomes of bone disease and anabolic treatment^13–15,35^. In our study, the statistical parameter sigma directly corresponds to the quantity of the calcium peak width in the BMDD. Therefore, the sigma parameter is potentially effective in evaluating bone development after bone disease treatment.

Skewness is a third-order statistical parameter and represents a measure of asymmetry in the distribution^16,21,22^. A positive skewness indicates that the distribution peak is shifted to the left and that the right tail of the distribution is longer than the left, while a negative skewness indicates the peak is shifted to the right and that the left tail is longer than the right. In this quantitative micro CT imaging study, a positive skewness indicates that the number of pixels with intensities lower than the average gray-scale intensity is greater than the number with intensities higher than the average gray-scale intensity, while a negative skewness indicates the opposite. Therefore, we can expect a negative quantity of skewness corresponding to bone of higher density in the specified ROI, indicating greater bone strength.

In statistics, kurtosis is a fourth-order parameter and represents a measure of peakedness, which is the functional shape of the peak in the distribution^16,21,22^. In micro CT images, a distribution with a positive kurtosis indicates that the shape of the peak is sharper relative to a Gaussian distribution and is called leptokurtic, while a negative kurtosis represents a peak shape that is relatively flat and is called platykurtic^16,21,22^. Therefore, a positive kurtosis suggests that the gray-scale intensities of bone images look more uniform than those with a negative kurtosis.

In our study, energy is an image feature parameter and represents uniformity, indicating variations of gray-scale intensities between pixels in images^16,21,22^. The maximum energy is one. A quantity of one for energy represents a uniform image in which the gray-scale intensities for all pixels are equal. Low energy suggests a relatively non-uniform image with a variety of pixel intensities. Therefore, we may observe large variations between pixels in bone images when the energy is low^16,21,22^. A large variation of gray-scale intensity in bone images may suggest a large discontinuity between pixels and the boundary, and a large discontinuity of bone density is easier to break when subjected to an external force^29,34^.

In physics, the quantity of entropy represents a measure of randomness or complexity for a physical state^36–38^. In CT images of cancer, entropy has been shown to be correlated with biological characteristics of tumours^39^. Our micro quantitative CT imaging analysis showed a decrease in entropy (from 4.10 to 3.85) from control mice to low-magnesium mice (*p* < 0.005).

The quantities of entropy calculated by Eqs 8–10 are 4.63, 5.23, and 5.43, respectively. The percentages of gray-scale intensities, that were below the 25th percentile in Eqs 8–10 are 0.55%, 9.69%, and 18.89%, respectively. We also observed that the linear correlation between entropy and that percentages of gray-scale intensities lower than the 25th percentile is high (*r*^2^ = 0.9221). Therefore, the entropy quantity extracted from micro CT images of bones was expected to be linearly correlated with the percentage of low-density bone in the specified ROI.

In a previous study of ultrasound Nakagami imaging, it was established that the Nakagami parameter is correlated with the scatter property of tissue material during the propagation of ultrasound transmission and is further correlated with tissue microstructure^40,41^. For CT imaging and x-ray physics, Rayleigh coherent scattering, the photoelectric effect, and Compton scattering are responsible for interactions between matters and x-rays of kV energy used in micro CT scanning^16^. In particular, the probability of the photoelectric effect is approximately proportional to the third power of the atomic number and probability of the Compton scattering is proportional to the physical density of the material. The most abundant inorganic element in bones is calcium (atomic number Z = 20), an element of relatively high atomic number, compared to that in soft tissues of organic compounds, hydrogen (Z = 1) and carbon (Z = 6). Therefore, a correlation between the Nakagami parameter and the composition of chemical elements in bone tissue in CT images was expected.

In this study, we showed that the Nakagami parameter was associated with the scatter of x-ray photons in micro CT imaging and bone tissue configuration. Bone heterogeneity is an essential quantity and is affected by the composition of different bone tissues. In particular, bone composition and therefore bone heterogeneity may be affected by the process of bone turn over, bone matrix dynamics, and bone development kinetics^13–15,42^. Therefore, the Nakagami parameter is a potentially useful quantity to evaluate different aspects of bone characteristics.

In previous bone research studies, a measure of BMDD was shown, and some quantities, such as peak width, were derived from BMDD^16,24,25,28,29^. The authors further investigated the dependence of BMDD on bone remodelling, mineralization kinetics, some bone diseases, and treatment outcomes. In our study, the mean was directly proportional to the BMD; sigma was a second-order statistical quantity and equivalent to the peak width of the BMDD; skewness was a third-order statistical quantity and an essential parameter to determine the shifting direction of the BMDD; Kurtosis was a fourth-order quantity and a parameter used to determine the shape of the gray-scale intensity distribution; and energy, entropy, and the Nakagami parameter were essential CT textural parameters correlated with bone heterogeneity. Therefore, our micro CT imaging approach of gray-scale intensity distribution for bone analysis extended the BMDD approach and could potentially be integrated with other imaging techniques, such as back-scattered electron imaging and Fourier transform infrared imaging^14^.

## Conclusions

We used a laboratory mouse animal model to perform a quantitative study on gray-scale intensity distributions that were acquired from micro CT images for bone assessment, and the results were shown to be effective. A set of seven quantitative parameters was obtained for comparisons between different groups of mice. The parameters of mean, sigma, skewness, energy, and entropy were effective in showing that the quality of cortical bone development was inferior in the low-magnesium group compared to that in the normal diet group. A linear correlation was established between the Nakagami parameter and BMD. We discussed potential interpretations of these parameters in bone analysis. BMD is typically used in existing approaches to bone diagnosis and bone treatment assessment. However, BMD is only an average quantity over a specified ROI and can be equivalently obtained from the gray-scale intensity distribution. In this study, we showed that the distributions of gray-scale intensities were very different, even though their means were identical. In particular, the relative percentage of low gray-scale intensities, indicating bone areas of low density, may suggest locations with a potentially high risk of fracture. Our study extended and added a new technical perspective to existing approaches to bone analysis.
